# An Effective Tri-Clustering Algorithm Combining Expression Data with Gene Regulation Information

**DOI:** 10.4137/grsb.s1150

**Published:** 2009-04-15

**Authors:** Ao Li, David Tuck

**Affiliations:** Department of Pathology, Yale University School of Medicine, New Haven, Connecticut 06510, U.S.A

## Abstract

**Motivation:**

Bi-clustering algorithms aim to identify sets of genes sharing similar expression patterns across a subset of conditions. However direct interpretation or prediction of gene regulatory mechanisms may be difficult as only gene expression data is used. Information about gene regulators may also be available, most commonly about which transcription factors may bind to the promoter region and thus control the expression level of a gene. Thus a method to integrate gene expression and gene regulation information is desirable for clustering and analyzing.

**Methods:**

By incorporating gene regulatory information with gene expression data, we define regulated expression values (REV) as indicators of how a gene is regulated by a specific factor. Existing bi-clustering methods are extended to a three dimensional data space by developing a heuristic TRI-Clustering algorithm. An additional approach named Automatic Boundary Searching algorithm (ABS) is introduced to automatically determine the boundary threshold.

**Results:**

Results based on incorporating ChIP-chip data representing transcription factor-gene interactions show that the algorithms are efficient and robust for detecting tri-clusters. Detailed analysis of the tri-cluster extracted from yeast sporulation REV data shows genes in this cluster exhibited significant differences during the middle and late stages. The implicated regulatory network was then reconstructed for further study of defined regulatory mechanisms. Topological and statistical analysis of this network demonstrated evidence of significant changes of TF activities during the different stages of yeast sporulation, and suggests this approach might be a general way to study regulatory networks undergoing transformations.

## Introduction

Clustering gene expression data generated has been intensively investigated in recent years and many efficient clustering algorithms, e.g. hierarchical clustering, have been presented and widely adopted in ordinary research. These methods use the whole data set for clustering, which assumes that the expression level of a gene is consistent throughout all experimental conditions. However, large scale data analyses demand more versatile ways to recognize subtle co-regulation associations among genes which may only present in specific experimental conditions. Bi-clustering is one promising methodology that addresses this problem by clustering genes and experimental conditions at the same time. Several bi-clustering algorithms have been proposed for this purpose, including CC,^1^ ISA,^2^ Bimax,^3^ and COSA.^4^ These methods vary significantly but most apply heuristic or stochastic algorithms with randomly selected initial seeds. The definition of a bi-cluster for each method can also be substantially different. For example, the CC algorithm defines a bi-cluster as a subset with high similarity expression patterns and measures it with a mean squared residue.^1^ For ISA and Bimax, the bi-cluster (also called “co-regulation module”) represents the significantly up-regulated or down-regulated genes under specific experimental conditions.^2^,^3^ The reason for different descriptions of a bi-cluster lies in the fact that there is no clear biological definition for it, no universal interpretation of the biological meaning, and no clear methods to validate proposed bi-clusters. In some studies, *p*-values calculated by the distribution of Gene Ontology terms^5^ or motifs detected with statistical significance^6^ were utilized to verify the inherent functional connections of the genes forming a bi-cluster. However, in general, there is no direct evidence that such genes are controlled together or commonly involved in some regulation mechanism. It is also feasible to interpret a bi-cluster by evaluating whether some of the genes can be mapped to a known regulation frame-work.^5^ However, it is not always possible to find a successful mapping for any one bi-cluster of interest. Thus, it would be desirable to extend the possible validation opportunities by identifying the direct regulators of the genes for specific conditions within clusters. By studying clusters generated by such a method, one may not only answer questions like “which genes perform quite similarly under some set of experimental conditions?”, but also answer such question as “what are these co-expressed genes controlled by?” or even “what is the regulatory mechanism and why it is condition-specific?”

In this study, we address these problems by introducing a new strategy which takes into consideration gene regulation information from additional sources. As an example, gene regulatory data from ChIP-chip experiments^7^ can provide evidence for direct binding of a gene’s promoter region by DNA binding proteins or transcription factors (TF). To demonstrate and evaluate our method we have therefore incorporated ChIP-chip datasets in combination with gene expression data before clustering analysis. Currently it is not feasible to perform as many whole genome ChIP-chip or Chip-seq experiments for all TFs of interest. To remedy this problem, we didn’t directly use the raw data from ChIP-chip results but adopted a regulator coefficient based on informational inference. Either a small TF-binding score or a low expression value of a regulator gene will significantly reduce this regulator coefficient and thus make it sensitive to variant experimental conditions. We refer to this measure as Regulated Expression Value (REV). The REV data space has three orthogonal dimensions: gene, experimental condition and gene regulator. None of the existing biclustering algorithms was designed to work in this space. We designed a three-dimensional clustering algorithm named TRI-Clustering which utilizes a divide-and-conquer strategy, and an Automatic Boundary Searching (ABS) algorithm that is able to detect statistically significant REV values that correspond to a three dimensional region in the whole data space named as a “tri-cluster”. Finally, we tested and analyzed the results and outcomes of the searching procedure in detail, demonstrating the method is valid and efficient.

This paper is organized as follows: first, results on synthetic data sets are analyzed to illustrate the effect of different parameters on the final results. Then we used the yeast sporulation data set to demonstrate how the algorithm works. We provide robust results and detailed analysis of the tri-cluster obtained from our method. Finally, we reconstructed the regulatory modules based on the tri-cluster and studied the centrality measurement of the network topology, and showed significant differences for the early and middle-late stages of sporulation.

## Material and Methods

Microarray experiments under a variety of conditions are widely available, and have been used to infer information about other eukaryotic cells. The data we adopted in this paper was provided by Chu et al^8^ and has been widely used for gene expression analysis in yeast. It contains more than 6200 genes and 7 different experimental conditions, including the early, middle and late stages of sporulation. The ChIP-chip experimental results come from the work of Lee et al^9^ who investigated the evidence for binding of more than 100 transcription factors to the promoter regions of 5535 genes. We found 5,349 genes with valid results on both ChIP-chip data and microarray sets, contributing to more than 86% and more than 96% of all the genes assayed by these experiments, respectively.

### REV data

Figure S1 demonstrates how the available information is used to construct a new three-dimensional data space. The first step of the procedure is to find the common genes analyzed in both ChIP-chip and microarray experiments (utilizing the ORF names annotated by Yeast Genome Database.^10^ The REV score indicates how a gene is affected by a TF in a certain experimental condition. For example, if the gene of a TF is highly expressed while at the same time, the expression of one of its possible target genes is significantly down-regulated (compared to other experimental conditions in the study), we should expect a high REV score assigned for this case. Thus, the REV score can be calculated by multiplying the normalized gene expression value with a regulation coefficient, formulated as:

(1) $$\begin{array}{l} REV(g,c,t)=\Theta (E_{N}(g,c)\cdot C_{R}(g,c,t))\\ =\Theta (E_{N}(g,c)\cdot E_{N}(t,c)\cdot \Psi (C(g,t))). \end{array}$$

Here the subscripts *g*, *c*, *t* denote each of the possible genes, conditions and TFs. *E_N_* (*t*, *c*) and *E_N_* (*g*, *c*) are the normalized gene expression values for TF gene and target gene, respectively. In this study, all microarray results in an experimental condition were standardized to a normal distribution with 0 mean and unit standard error. *C*(*g*, *t*) is the ratio from ChIP-chip data and Ψ() is a linear-scale function which maps all ratio values for one experiment into the range of 0 to 1. When a gene is not regulated by a TF (low ChIP-chip value) or not differentially expressed (low microarray value), the corresponding REV scores will be close to 0. On the contrary, when the absolute value of a REV is quite large, it suggests that the gene is activated or suppressed in this condition. Finally, we applied a tail-cutting function Θ with a threshold 0.01 to eliminate trivial values for further clustering and analysis, which simply sets all REV scores below this pre-defined threshold to 0.

### Clustering algorithm

**Figure 1:**
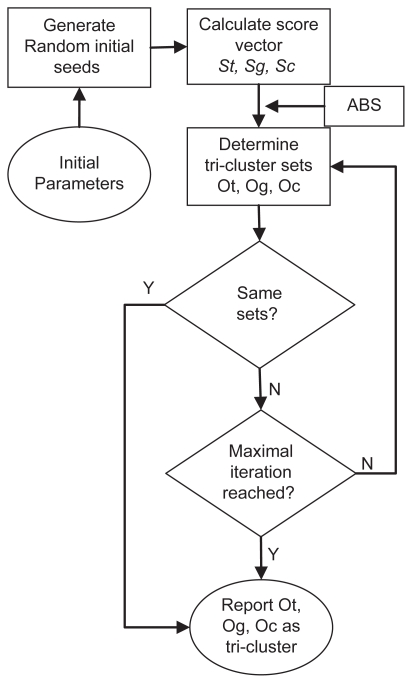
Flowchart of the TRI-Clustering algorithm.

We introduce an efficient algorithm named TRI-Clustering (Three-dimensional REV Iterative Clustering Algorithm) to explore REV data. The outline of this algorithm is displayed in Figure 1. Similar to previous bi-clustering algorithms such as ISA^2^ or SAMBA,^3^ it employs a heuristic searching method with randomized initial tri-cluster and parameters. It will stop at a local optimization if the maximal number of iterations is reached (in this study, this number is set to 100). The algorithm starts with these randomly generated seeds. For each iteration, it exhaustively calculates the score of every possible two dimensional region in the whole subspace. For example, given a REV data space with *N* genes, *M* experimental conditions and *L* TFs, calculation of the TF score can be expressed as:

(2) $$\begin{array}{l} {St}_{i}=\left(\sum_{j=1}^{N}\sum_{k=1}^{M}|REV(j,k,i)|\right)/\left(\sum_{j=1}^{N}I(Sg_{j})*\sum_{k=1}^{M}I(Sc_{k})\right)\\ -\left(M*N-\sum_{j=1}^{N}\sum_{k=1}^{M}I(|R(j,k,i)|)\right)*\delta p,i=1KL \end{array}$$

where *I*() is an indication function to calculate the number of zeros or negative scores. *Sg*, *Sc* are further defined as the score vectors for gene and experiment scores. They are calculated similarly as *St* in equation 2. To avoid too many zero values in the final results, we used a punishing coefficient *δp* (0.01 in this study) for every 0 value in a tri-cluster. The time complexity of Equation 2 is *O* (*Ng* * *Nc*), where *Ng* is the number of genes in the data sets and *Nc* is the number of experimental conditions. Thus, the time complexity for the computation of the TF score vector *St* will be *O* (*Ng* * *Nc* * *Nt*) where *Nt* is the number of TFs. The ABS algorithm is then used to automatically find an appropriate threshold as the boundary of normal and outlier sets. In the same way, Sg and St are all recalculated and all three outlier sets are compared with those found in previous runs. The algorithm will continue running until all outlier sets are the same or a maximal number of iterations is reached.

### Automatic boundary scanning algorithm

An important task for TRI-Clustering during the searching procedure is to identify significant averaged REV scores (outliers), which are distinct from the background distribution, as described in Equation 2, These outliers are related to a subset of genes/TFs/conditions that contribute to a tri-cluster. A fixed threshold is not desirable as values calculated in different iterations may vary dramatically. In the ISA algorithm, the standard variance for all scores (including outliers) is used, which clearly may exaggerate the difference between normal scores and make it harder to identify outliers.^2^ Kloster et al^5^ suggested a variable threshold accounting for more than 70% of all scores to eliminate the effect of outliers. However, a pre-defined percentile as a threshold lacks flexibility and may not be a good general estimation of the number of normal scores. Here, we present a new algorithm that can statistically detect the boundary between the outliers and normal scores. The ABS algorithm (outlined in Supplementary Fig. S2) starts from an initial parameter *T* (for specificity, the initial value of *T* is set to 5 or more) and uses the whole set as the set for normal scores in the first iteration. Then outliers are detected and removed from the normal set. For each run, if no outliers can be found by ABS, *T* is updated by adding a small step size *Delta*. Both the outlier and normal sets are determined by the value of another variable (*B*), which is calculated as the mean of the current outlier and its standard variance multiplied by *T*. If the outlier sets for two continuous iterations are exactly the same, then the algorithm will stop and report outlier sets and current *B* Value. In one iteration of the TRI-Clustering algorithm, the complexity of the ABS algorithm is *O*(*Ng* + *Nc* + *Nt*). So the time complexity for the whole procedure will be *O*(Ng*Nc*Nt) + *O*(*Ng* + *Nc* + *Nt*).

### Data normalization

By using the data described in the material section, we found 5,349 genes have valid results on both ChIP-chip data and microarray sets, contributing to 86.2% and more than 96% of all the genes assayed by the two experiments, respectively. Furthermore, we normalized these genes within each one of the experimental conditions to a normal distribution with 0 mean and unit standard variance. To scale the ChIP-chip data set, following the work of Lee et al^9^ we first used a simple “present or not” assignment to every gene under each one of the TF binding experiments with a P-value threshold of 0.001. However, the results are clearly not precise with an arbitrary cut-off score, for at least two reasons. First, a score little less than 0.001 will be treated differently than one slightly bigger than 0.001, while in fact the difference may be trivial. Second, the distance of scores from the threshold is not taken into account, with values above the threshold all scaled to 1, and all below the threshold assigned to 0. To solve this problem, we used the linear-scaled ratio values of ChIP-chip experiments within the range of 0 to 1. Given a series of scores *R* = {*R_i_*, *i* = 1k … *n*} the formulation of the linear scale is:

(3) $$R_{j}^{\prime }=\frac{R_{i}-\text{min}(R)}{\text{max}(R)-\text{min}(R)}$$

### Algorithm analysis

**Figure 2:**
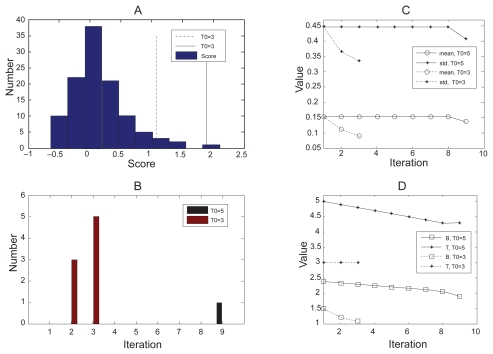
Experimental results by ABS algorithm. **A**) Histogram of *St* scores. Solid line represents boundary for *T*0 = 5, dashed line is the boundary for *T*0 = 3. **B**) The numbers of outliers found by ABS during each iteration. **C**) Changes of mean and standard variance. Solid lines for *T*0 = 5 and dashed lines for *T*0 = 3; cycle for mean values and star for standard variances. **D**) Changes of T and B Values during execution. Solid lines for *T*0 = 5 and dashed lines for *T*0 = 3; square for B Values and star for T values.

We selected a series of *St* scores during the execution of clustering and report how the ABS algorithm works. A typical initial *T* value (3.0) and a large initial value (5.0) were evaluated to see how they affect the results. Either of these thresholds is sufficient to detect outliers (Fig. 2A shows the histogram of the scores), with boundary values of 1.89 (solid line) for *T0* = 5 and 1.23 for *T*0 = 3 (dashed line). It is easy to conclude that the bigger *T*0 is more specific for outliers that are far away from the center, while with the more sensitive value of 3.0, we can find outliers that are harder to distinguish visually, but which can also be detected by ABS. Figure 2B illustrates the outcome for each iteration. With *T*0 = 3.0, the ABS algorithm picked outliers at the first step and converged in 3 iterations. With the larger initial value, the algorithm does not converge until 9 iterations (the step size in this procedure was set to 0.1). Also, if big enough (usually >3), *T* score is not sensitive to the outliers generated in each iteration and thus does not have significant effect on the tri-clusters found by the algorithm. Figure 2 also shows the changes during execution of mean and standard variance (Fig. 2C), and the T and B values (Fig. 2D), We can conclude that the ABS algorithm, though varying with different parameters, always seeks a minimal variance within the normal set and a boundary as close to the center of scores as possible in order to identify more outliers.

**Figure 3:**
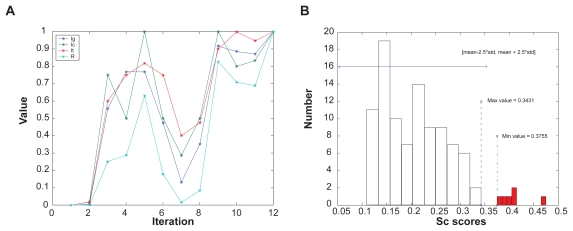
Analysis of the TRI-Clustering algorithm. A) Changes of similarity scores during TRI-Clustering execution. B) Histogram and detailed results of Sc scores.

In order to provide some understanding how the clustering algorithm converges, we display output information of the TRI-Clustering algorithm during execution as an example in Figure 3. In each run, the subsets of genes, conditions and TFs detected were compared with the results for the previous time and the numbers of the unchanged elements were recorded, For convenience, a similarity score to describe the similarity of two sets returned by the TRI-Clustering algorithm in subsequent iterations (see Fig. 1) is defined below:

(4) $$I_{ij}=\frac{\left||O_{ij}IO_{i-1j}|\right|}{\left||O_{ij}YO_{i-1j}|\right|}$$

where *i* is the number of iterations and *j* ∈ {*g*, *c*, *t*}. Furthermore, the overall similarity score of a tri-cluster can be derived as:

(5) $$R_{i}=\underset{j\in \{g,c,t\}}{\prod I_{ij}}$$

All these scores calculated at each iteration are displayed in Figure 3A. All values start from a pre-defined 0 value and usually increase very little for the first several iterations (here iteration 2 only) as the random initial subsets can hardly be optimal which will lead to dramatic changes. Gradually, the algorithm will heuristically detect some tri-clusters which yield larger similarity scores (iteration 3, 4, 5) and may reach a local maximum (iteration 5). At this point, if the stopping criterion is satisfied (i.e. *I_ig_* = *I_ic_* = *I_it_* = *R_i_* = *1*), TRI-Clustering algorithm will stop and return the current cluster. Otherwise, it will continue to search in the whole data space, which means usually changing from a small, subtle lattice to a large, obvious one. During this procedure, the scores will commonly decrease as further search continues since the identification of another tri-cluster can not be perfectly achieved in only a single iteration (iterations 6, 7). However, if the new tri-cluster is good enough, all values will increase and finally converge (iterations 8–12) to a stable tri-cluster.

Furthermore, to study how well the cluster can be distinguished from the background data, we examined the distributions of scores belonging to subsets of a tri-cluster and compared them with those of normal scores. The *Sc* scores of a tri-cluster are demonstrated in Figure 3B. The white bars in the histogram represent the distribution of normal scores while the red represent outliers. The maximal value of the normal set is 0.3431 and the minimal value of the outlier set is 0.3755. These two sets are clearly separated, which indicates the tri-cluster is also very distinct in the *St* dimension. The *T* value at the end of iterations is 2.5, which can perfectly detect and separate the outliers from background.

## Results and Discussion

### Effect of punishing coefficient

Non-zero values are usually sparse in REV data. Calculating the scores along one dimension (e.g. *St* along the TF dimension) in cooperation with a negative value *δp*, is equivalent to solving the mathematical average over a 2-dimensional space in which all zero-values are replaced with *δp* (Fig. S3A). Punishing coefficients are useful to enable the TRI-Clustering algorithm to better identify sub-regions of interest. However, an extremely negative punishing value can nullify the results of non-zero values and reduce the signal-noise ratio (Fig. S3B), as scores obtained this way are far smaller than zeros and with a much larger variance due to the large absolute value of *δp*. Furthermore, a moderate punishing value makes it easier for the heuristic algorithm to “climb over” a high “potential energy” region (Fig. S3C) to reach a better solution. With an extreme punishing value, it may only detect some local optimal clusters that are close to the regions of random initial seeds. We will provide detailed analysis on its effect in the synthetic data section.

### Results on synthetic data sets

The effect of the punishing coefficient was investigated in order to find an optimal value for further study. First, we start with the simple case by considering only one TF because it is easy to understand and the results can be compared with other bi-clustering algorithms. Within a manually-generated data matrix with 400 genes and 400 conditions, we assume there exists a true rectangular cluster with the size of 100 (sampled from normal distribution N(0.5,1)). Also, to mimic real REV data, a false positive cluster was also generated which is statistically distinguishable from the true cluster (sampled from N(0,1)). All other values in the data matrix were set to 0, as would be the case commonly with REV data. We tested the sensitivity and specificity of the algorithm (over 100 repetitions) with six coefficients with different absolute values: 0, 0.001, 0.01, 0.1, 1, 10. (The results are displayed in Supplementary Table S1). T scores for genes, conditions and TFs, were set to 0.1, 0.1 and 1, and the parameter for initial seeds was 0.05. In this test, without any specific punishment for zero values in the cluster found by the algorithm, a perfect sensitivity can be achieved which means the TRI-Clustering algorithm can successfully detect the true cluster. When zero values in the false positive cluster, were also included in the results, the specificity was reduced to 80.1%. At the same time, increasing the punishing coefficient can successfully increase specificity to 98.1%. However, the algorithm will not benefit from extreme large punishing scores as described previously. When set to greater than 1, the overall performance significantly decreased. Thus, for the further computation work in this study, the punishing coefficient was set to a moderate value of 0.01.

**Figure 4:**
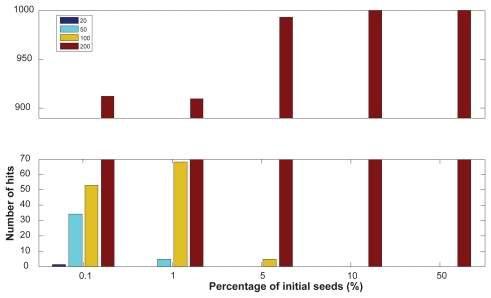
Histogram of the clusters with different sizes found by the algorithm on synthetic data.

We explored the effect of the number of initial seeds on the results produced by the TRI-Clustering algorithm by simulating a new synthetic set of tri-clusters with different sizes. In total four tri-clusters of size 20, 50, 100 and 200 were generated by sampling from a normal distribution with mean 1 and standard deviation of 0.1. Then we tested the performance using different percentages of total genes/conditions/TFs as initial seeds. In total, 5 different percentages, 0.1, 1, 5, 10 and 50, were used and initial seeds were randomly generated with uniform distribution. The program was run 1000 times for each parameter. The final results are shown in Figure 4. As is evident, the biggest cluster dominated the results no matter what initial seeds were chosen. Our method exclusively detected the biggest cluster, especially when a large portion of data was used for the first iteration, (e.g. 10% or 50%). With a smaller percentage, 5%, the algorithm found 5 hits of the second largest clusters in 1000 times. With a coefficient 0.1% (i.e. an average of only 1 gene/condition/TF selected initially), all four clusters can be successfully identified including the smallest cluster with size of 20. This is because the heuristic searching algorithm used by TRI-Clustering makes the optimal solution dependent on initial seeds, especially when there are multiple tri-clusters in the data. In the above example, when there is a large, significant tri-cluster, more initial seeds will be selected within the large cluster if the number of initial seeds increase, which will lead the algorithm to have much more chance to find this large cluster instead of subtle clusters. Therefore, a smaller number of initial seeds should be used if the TRI-Clustering algorithm is employed to detect subtle clusters. Alternatively, a higher number of initial seeds should be included to obtain more robust results. Also, if extremely large tri-clusters are found, they should be removed from the data and the algorithm should be rerun to detect more tri-clusters.

Finally, before working on real data, synthetic REV scores were generated to validate our method. We used different ways to create these data. First, we would like to see how it works compared to published bi-clustering methods. Two algorithms, Bimax and ISA, were selected as they have similar definitions of a bi-cluster to the one we use in this study. To make such a comparison reasonable, we used only one TF and thus the REV data reduced to two dimensions and behaves similarly to normal microarray data. We generated 500 genes from 40 experimental conditions, and assumed only 50 genes were controlled by the TF that worked actively in 10 conditions. The expression values of the (single) actively regulating TF were sampled from a normal distribution with mean 1 and a pre-defined standard variation, which we varied in the simulations. Other TFs were sampled from a normal distribution of the same variation but with the mean of 0. With the hypothesis of exponential effect of a factor on the target, we expect a linear relationship between the corresponding expression values after log transformation. Therefore, a gene regulated by the TF was sampled from the expression of the TF plus a unit normal distribution of the same deviation variation). Unregulated genes were generated from the unit normal distribution only. For the synthetic ChIP-chip data, the active status and inactive status were sampled from normal distributions of 1 and 0, with a standard deviation of 0.1. Finally, to mimic false positives/false negatives in real ChIP-chip data, we randomly shuffled these data by a certain percentage (either 10% or 20% in separate simulations). We tested a series of standard deviations from 0.1 to 0.5, ran each procedure 20 times and averaged the final results. The implementation of the bi-clustering algorithms for comparison is from the BicAT toolbox.^11^ The values of sensitivity (*sn*), specificity (*sp*), and over-all accuracy (*acc*) are defined as the percentage of correctly identified TF-gene pairs and are shown in Table 1. With small standard deviation in the simulated data, all three algorithms worked well. As the microarray data variation increases, the performance of all methods decreases, especially when the standard variation reaches 0.5. TRI-Clustering algorithm performs comparably to other methods and even better in some scenarios, e.g. when standard deviation equals 0.1. The results indicate that incorporating prior information about gene regulation, the TRI-Clustering algorithm can detect bi-clusters more accurately. However, it should be pointed out that the quality of prior information is critical to the prediction performance. As is shown in Table 1, the accuracy when 20% noise was added through permutation (more false positives/false negatives in simulated ChIP-chip data) was much less those obtained with only 10% permutation.

### Interpretation of tri-clusters

We used the yeast sporulation data set to examine in detail the behavior of the TRI-Clustering algorithm. The initial seed ratio was set to 0.3. T scores for both TFs and conditions were 3. The T score for genes was fixed at 6 as there are more than 5,000 genes included and only the most significantly changed genes are desired. The punishing coefficient used here was 0.01. One resultant tri-cluster is illustrated as an example. It contains 449 genes significantly differentially expressed; 274 genes in the tri-cluster were up-regulated during this period and 175 were down-regulated. These induced genes were compared with list of 137 genes that were previously reported to be associated with sporulation,^8^ and 62 of them were found in the tri-cluster. For the other 25 genes involved in metabolism in this process,^8^ all were included in this cluster.

**Figure 5:**
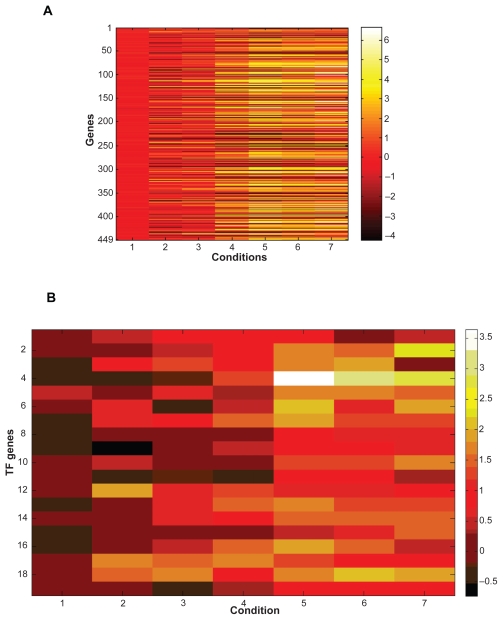
**A**) Expression values of the 449 genes in the tri-cluster. **B**) Expression values of the 19 TF genes in the tri-cluster.

In total, there are seven samples at time points 0, 0.5, 2, 5, 7, 9, 11 hours. The subset of conditions selected is the latter 4 time points. We drew the heatmap of the microarray data for the 449 genes over all conditions as well as the expression values of the 19 TF genes (shown in Figs. 5A and B). Apparently, the behavior of all these genes at baseline is far away from the other conditions. For the early stages of sporulation (condition 2 and 3), although a small number of genes is differently expressed, such as IME4 (up-regulated), ZIP2 (up-regulated), ENO2 (down-regulated) etc, the overall pattern in the early period is quite different from the middle or late stages. Also the 19 TF genes were up-regulated in the middle and late stages of sporulation (Fig. 5B). For further analysis, the REV values for different TFs were averaged and then compared by experimental conditions (Supplemental Fig. S4). It is apparent that conditions within either group (Group 1: condition. 1, 2, 3, Figure S4.a; Group 2: condition. 4, 5, 6, 7, Fig. S4.b) perform quite similarly to each other but quite differently from those not in the same group. For example, the correlation coefficient (CC) for condition 3 and condition 2 is 0.680, but with condition 7, the score is only 0.003. The averaged correlation coefficient for all pairs of conditions present in the cluster is as high as 0.774.

**Figure 6:**
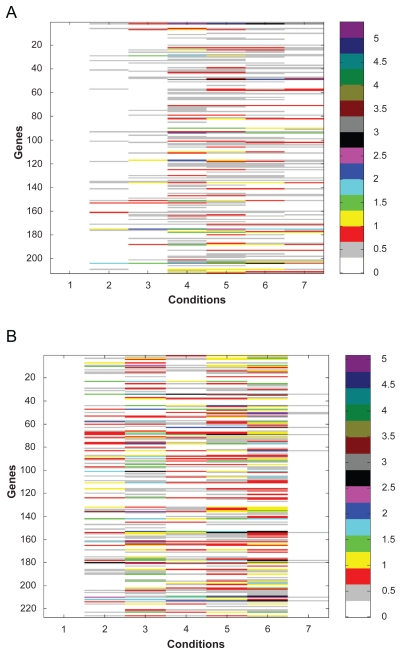
**A**) Regulation profile of IME4 for yeast sporulation **B**) Regulation profile of FKH1 for yeast sporulation.

Using the concept of REV score, we can access regulation behavior of a TF under different experimental conditions. We built the regulation profile for each TF from the REV data and showed the profile of IME4 and FKH1 in Figure 6. IME4 is a factor identified as a clone that enhances RES1–1-dependent spr3-lacZ expression and reduces or abolishes IME1 and IME2 expression and sporulation when disrupted.^12^,^13^ By analyzing the REV values of all genes regulated by IME4, it is observed that most of the significant regulation events happen after the 2 hour time point (Fig. 6A), which suggests for most cases, IME4 acts as a middle and late stage gene regulator. Although most of the TF profiles are similar to this one, for some specific TFs, the profile patterns are quite different. For instance, the profile of FKH1 (shown in Fig. 6B) shows the conditions that were affected by FKH1 are focused on the early to middle stages instead of the first and last condition. This verifies the conclusion that FKH1 is induced early during sporulation and is involved in some transcriptional cascades for early and middle genes.^8^,^13^

Moreover, the results were validated by comparison with the Open REGulatory ANNOtation database (ORegAnno), an open database for the curation of known regulatory elements from the scientific literature.^14^ We downloaded the data files with all known TFs with target gene information in yeast and parsed them into more than 4,000 TF-gene pairs. We observed that 86 pairs in the tri-cluster can be verified in the ORegAnno database (detailed information can be found in Table S1). To test if this result is statistically significant, we performed a procedure that randomly selected tri-clusters with the exact same sizes as we got from TRI-Clustering algorithm, and calculated how many TF-gene pairs can be detected in OregAnno. We repeated this procedure 10,000 times and the *p*-value associated is 0.009, suggesting a significant abundance of true TF-gene interactions found in the result of our method. Finally, we used TRANSFAC^15^ to investigate if there are any over-represented TF motifs in the promoter sequences of the genes in the tri-cluster. Up to 5,000 base-pair promoter sequences were downloaded from UCSC genome database and searched against the TRANSFAC motif database. The CLOVER program^16^ was employed to detect any *Cis*-element overrepresentation. The promoter sequences of more than 5,000 yeast genes were generated and used as a “background” in CLOVER. We identified five motifs resident in the promoter sequences: F$ABF1_01, F$HAP234_01, F$LEU3_B, F$RAP1_C and F$REB1_B, of which F$HAP234_01 and F$RAP1_C are significantly over-represented with *p* values 0.009 and 0.008. All these results provide strong supportive evidence of gene regulation events inferred from the tri-cluster.

To further pinpoint the regulation mechanisms of these TFs, we tried to rebuild corresponding parts of regulatory modules from the tri-cluster. Here a threshold 0.1 was used to discretize the values in TF profiles described above. Network centrality was adopted in this work to describe the topologic character of condition specific networks.^14^ One formulation of centrality is the out-degree that is the number of genes regulated by a TF. The out-degrees for all 19 TFs in the tri-cluster are shown in Table 2. It is easy to find the maximal values for TFs which only appear in the subset conditions of the cluster. At the same time, the average out-degrees of TFs in the middle-late stages are significantly higher than the early stages, which can serve as an indicator for grouping different experimental conditions. The averaged out-degrees, when all TFs are taken into account, didn’t change much during the entire sporulation period (Table S2). The results suggest that during the middle and late stages of sporulation, these TFs are highly activated and have more influence on target genes.

**Figure 7:**
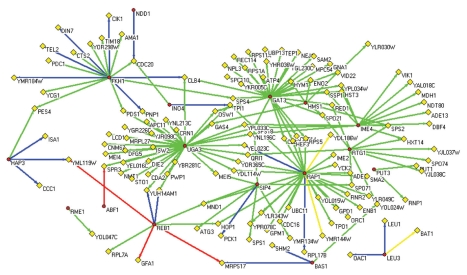
Regulation network reconstructed from tri-cluster. Red circles denote genes under regulation and yellow diamonds denote TF genes. Green arcs are significant TF-gene regulations, yellow arcs are significant TF-gene regulations with motif found in promoter sequences, blue arcs are significant TF-gene regulations verified by OregANNO, red arcs are significant TF-gene regulations with motif information and can be found in OregANNO database.

Finally, a regulatory network was reconstructed based on the gene regulatory information from the tri-cluster. We selected significantly regulated TF-gene pairs (with a threshold of 0.5 for the mean REV scores over all conditions in the cluster) and integrated them into a whole regulation network (shown in Fig. 7). This network provides a broad picture of gene regulation occurring in the tri-cluster. For example, we can observe in the middle and late stage of yeast sporulation, there are three target genes: OAC1, LEU1 and BAT1, controlled by LEU3. All of these can be verified by either TRANSFAC or OregANNO. We can also identify more TFs with validated target genes and some novel ones that haven’t been verified yet. A typical example is REB1, which has three target genes (YML119W, MRPS17 and GFA1) with strong evidence from both information resources and two more targets: YUH1 and MAM1 can be found in OregANNO database. Also, there are some new targets found without any previous knowledge, for example, HOP1, which is also regulated by another TF RAP1 and suggests a possible regulation mechanism by these two TFs. Moreover, there are other important TFs acting as a hub node in the regulation map, such as IME4, GAT3, UGA3, etc. Although we can not verify any target genes associated with them (sometimes due to the absence of known targets), but the functionaries of most of them have been recognized by previous studies. For example, IME4 is known as an important regulator for middle and late sporulation and UGA3 is a transcriptional activator necessary for gamma-aminobutyrate (GABA)-dependent induction of GABA genes like UGA4, just as shown in the figure. Also they share a lot of target genes as well as with other TFs in the network, indicating intimate interactions and cooperative functions among these TFs and the complexity of the whole gene regulation network.

## Conclusion

In this study, we extend the original two dimensional microarray data to a three dimensional REV space that incorporates TF binding information from ChIP-chip experiments, expression levels of TFs and expression levels of regulated genes. To explore this kind of data, we extended previously described two dimensional clustering approaches and developed an efficient and robust method: TRI-Clustering algorithm with a sub-algorithm for automatic threshold detection. We provided detailed results and analysis of a tri-cluster and the associated regulatory network.

The bi-clustering concept provides for identification of sets of genes that are condition specific, and may not be found by classical clustering which operates on all experimental conditions. However, bi-clustering only leads to the point of identifying co-expressed genes which then leaves the task of predicting or explaining the regulatory mechanisms as a further interpretive step. The tri-clustering concept which we propose here provides an explicit representation of the regulatory effects in the TF-gene network and also clearly identifies transitions in the network from condition to condition which are implicit in the boundaries of the identified tri-clusters.

The meaning of this REV space involves the intersection of three orthogonal planes represented by familiar two dimensional matrices. The T-G (transcription regulatory factor-target gene) matrix defines a connectivity network in which the potential for a regulatory factor to influence a gene is present for at least some conditions. The links in this transcription factor connectivity network indicate the potential for a regulatory factor to influence the expression of a gene through transcriptional or post-transcriptional means. In the case of DNA binding proteins, this indicates in the simplest case a potential DNA binding site in the promoter, or other regulatory region of a gene, and the potential for occupancy under some biological conditions relevant to the analysis at hand. For a microRNA similarly this might indicate similarly a complementary *cis*-regulatory region, or other target for a hairpin structure. Alternatively, for gene silencing, this connectivity network might indicate that DNA methylation of the promoter occurs under some relevant conditions. And, for chromatin modifications, this might indicate that the methylation or acetylation of a particular histone site may occur indicating silencing or activation under the relevant conditions. The G-C matrix is the matrix for the gene expression for the entire universe of genes under the complete conditions in which the hybridization was performed, while the T-C matrix (transcription factor-condition) represents the specific condition activity/influence of particular regulatory factors.

Unlike bi-clusters extracted from microarray data alone that have no explicit information about regulation, a gene in a tri-cluster always is controlled by at least one regulator. At the same time, compared to traditional models inferred from ChIP-chip data, it has novel information about the specificity of experimental conditions. From the view point of informatics theory, the regulation evidence from ChIP-chip experiments can be regarded as prior knowledge for general conditions. But it does not guarantee that under all circumstances, these rules can apply. By adding new sources of data specifying activity under different conditions, we can refine the existing information about transcription regulation by machine learning algorithms.

Vast amounts of detailed gene regulatory information are already or will become available in the near future, including transcription factor regulatory interactions, transcriptional or post-transcriptional events related to small RNAs, sense-anti-sense in-teractions, or epigenetic activities including DNA methylation, histone modification, and chromatin remodeling. Novel approaches to detect subtle and complex regulatory events among various factors are needed. One intriguing question about REV data in higher dimensional spaces is the capability of reflecting all these kinds of regulation influences, and extending related algorithms to analyze and integrate all such information.

## Supplementary Information

Figure S1 Illustration of the mapping procedure by interpolating microarray data matrix with transcriptional binding matrix. **A**) Data for CHIP-Chip and microarray experiments are normalized before calculation. **B**) The new data space has 3 orthogonal dimensions (i.e. Genes, Conditions, TFs) and all values are obtained by Equation.

Figure S2 Pseudo code for ABS algorithm.

Figure S3 Illustration of the effect of punishing coefficient. **A**) The same region in data space with different punishing coefficients. Center block (green) is a region for clustering. **B**) Effect on score distribution. Big value of punishing coefficient (lower) will reduce Signal-noise ratio and make a cluster harder to detect. **C**) Effect on searching algorithm. Small punishing value (upper) will make the heuristics possible to overpass a high “potential energy” region from a local minimum and find the more stable and obvious cluster.

Figure S4 Averaged REV scores for different experimental conditions.

Table S1 Comparison of prediction performances with different punishing coefficients.

**Table:** 

Punishing-Coefficient	0	0.001	0.01	0.1	1	10
Specificity (%)	80.1	80.4	81.6	98.1	85.0	75.0
Sensitivity (%)	100	100	100	100	98.3	97.2

Table S2 Detailed information about out-degrees for different stages of yeast sporulation.

**Table:** 

Condition	1	2	3	4	5	6	7
Mg/Mt	21	66	71	127	152	151	125
Mg/~Mt	27	20	12	7	5	6	11
~Mg/~Mt	258	110	42	9	4	6	18
All/All	266	183	121	100	111	110	102

Mg: list of genes included in the cube cluster, ~Mg: list of genes not included in the cube cluster, Mt: list of TFs included in the cube cluster, ~Mt: list of TFs not included in the cube cluster, All/All: all genes and all TFs examined in this study.

## Figures and Tables

**Table 1:** Performance comparison of TRI-Clustering and other methods.

	Std = 0.1			Std = 0.2			Std = 0.3			Std = 0.5		
	*Sn*	*Sp*	*Acc*	*Sn*	*Sp*	*Acc*	*Sn*	*Sp*	*Acc*	*Sn*	*Sp*	*Acc*
TRI-Clustering (10% permutation)	99.5	100	99.9	86.4	100	99.7	94.0	99.7	99.6	37.6	99.0	97.5
TRI-Clustering (20% permutation)	92.0	100	99.8	82.8	100	99.6	92.0	99.7	99.6	35.3	99.1	97.5
BiMax (threshold = 0.7)	88.2	100	99.7	37.2	100	98.4	26.4	100	98.2	21.6	100	98.0
ISA (t_g = 1, c_g = 1)	99.0	97.8	97.8	90.0	97.9	96.8	90.0	97.6	97.4	40.0	95.3	93.9

**Table 2:** Centrality Changes (measured by Out-degree) of different stages in yeast sporulation.

TFs	Condition 1	Condition 2	Condition 3	Condition 4	Condition 5	Condition 6	Condition 7
ABF1	0	73	93	**120**	106	40	80
BAS1	6	9	48	86	107	102	**114**
FKH1	0	143	182	171	201	**212**	59
GAT3	0	0	0	**226**	245	241	228
HAP3	97	16	59	59	135	**143**	131
HMS1	0	99	0	126	**180**	151	158
IME4	0	85	115	176	**180**	165	146
INO4	0	65	0	101	**155**	148	123
LEU3	0	0	25	56	65	**79**	46
MET4	6	87	0	51	**189**	187	179
NDD1	69	0	0	0	91	**105**	27
PUT3	3	153	149	**191**	163	124	115
RAP1	0	108	177	**222**	219	217	199
REB1	78	26	92	142	159	**168**	159
RME1	0	30	17	54	48	**125**	119
RTG1	0	60	95	219	**222**	217	90
SIP4	26	137	157	**192**	168	161	145
UGA3	105	133	144	152	165	**180**	160
ZMS1	0	33	0	71	91	**110**	94
TOTAL	390	1257	1353	2415	**2889**	2875	2372
