# Supplementary figures and images for: An Effective Tri-Clustering Algorithm Combining Expression Data with Gene Regulation Information
Source: Gene Regul Syst Bio. 2009 Apr 15;3:49–64. doi: 10.4137/grsb.s1150 (PMC2758278; doi:10.4137/grsb.s1150)

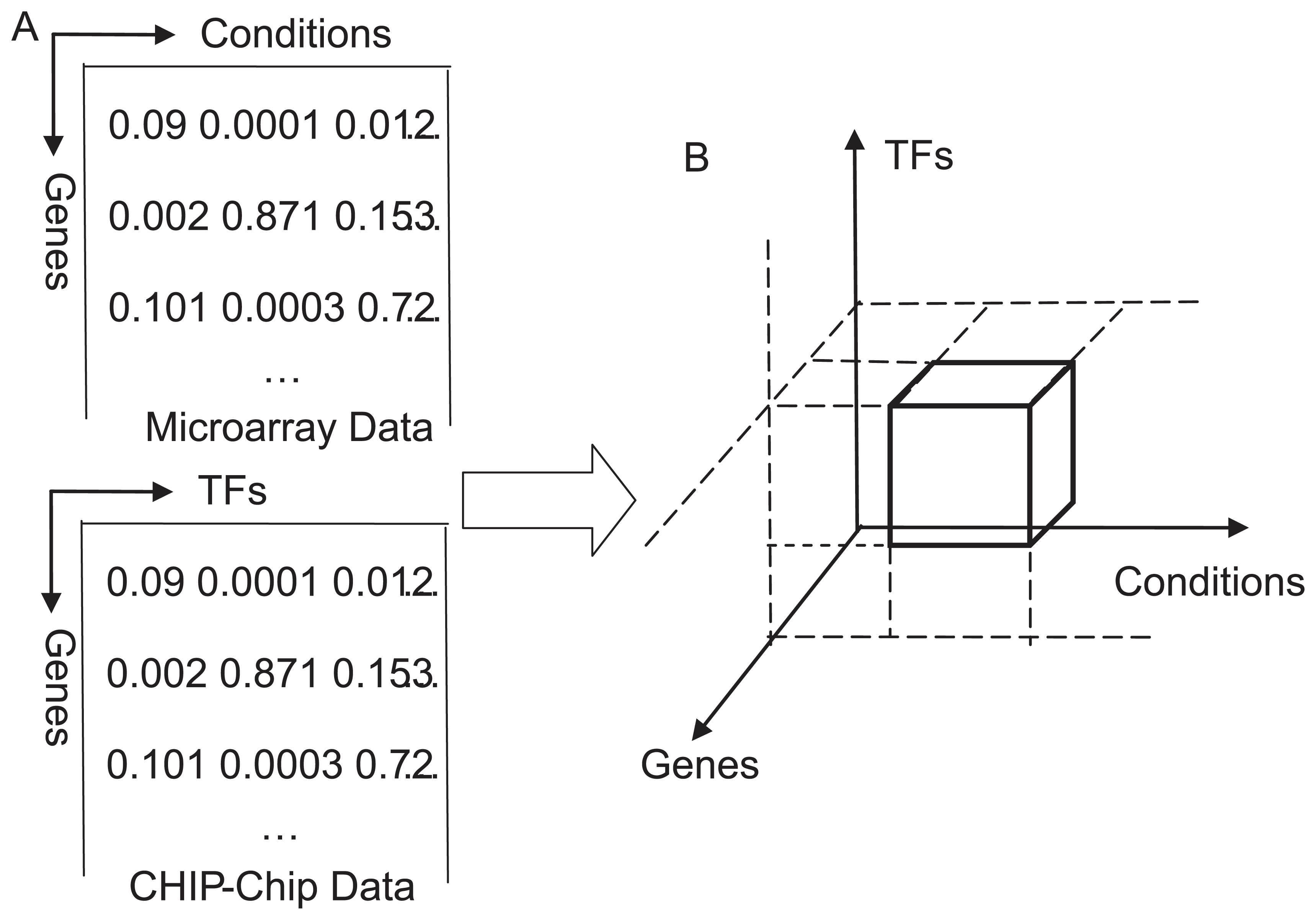

Supplement: Figure S1 — Illustration of the mapping procedure by interpolating microarray data matrix with transcriptional binding matrix. A) Data for CHIP-Chip and microarray experiments are normalized before calculation. B) The new data space has 3 orthogonal dimensions (i.e. Genes, Conditions, TFs) and all values are obtained by Equation. [file grsb-2009-049f8.tif]

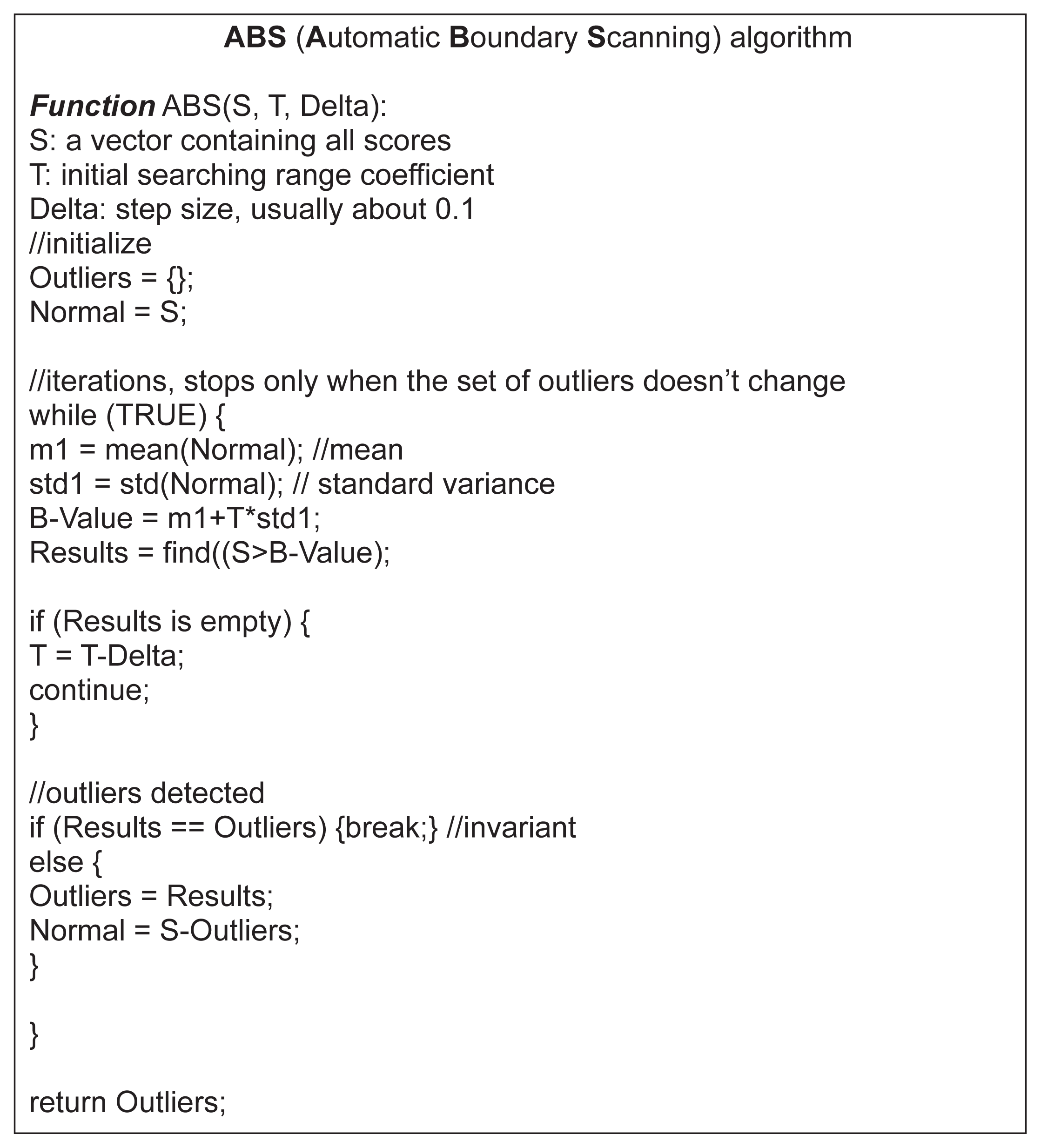

Supplement: Figure S2 — Pseudo code for ABS algorithm. [file grsb-2009-049f9.tif]

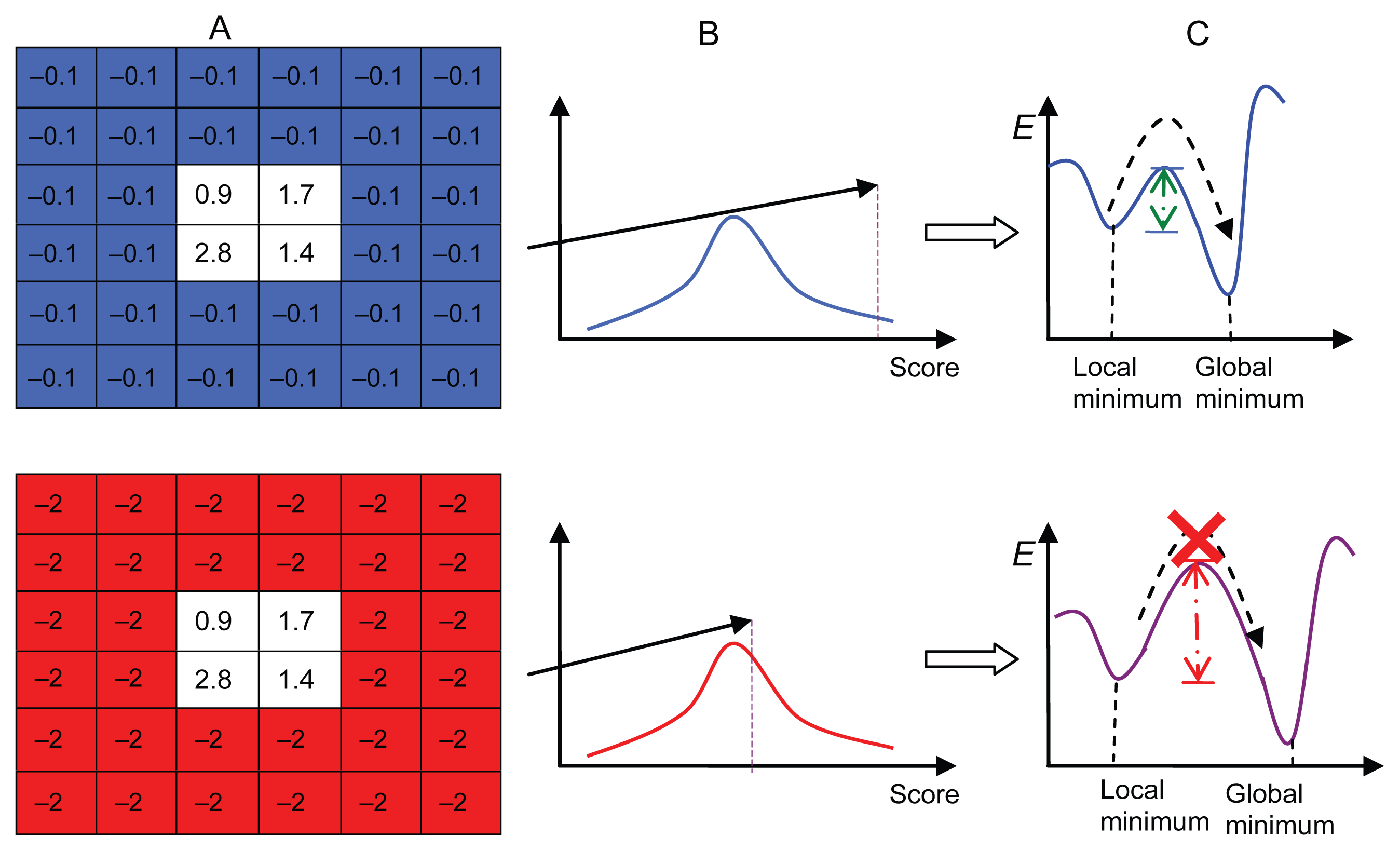

Supplement: Figure S3 — Illustration of the effect of punishing coefficient. A) The same region in data space with different punishing coefficients. Center block (green) is a region for clustering. B) Effect on score distribution. Big value of punishing coefficient (lower) will reduce Signal-noise ratio and make a cluster harder to detect. C) Effect on searching algorithm. Small punishing value (upper) will make the heuristics possible to overpass a high “potential energy” region from a local minimum and find the more stable and obvious cluster. [file grsb-2009-049f10.tif]

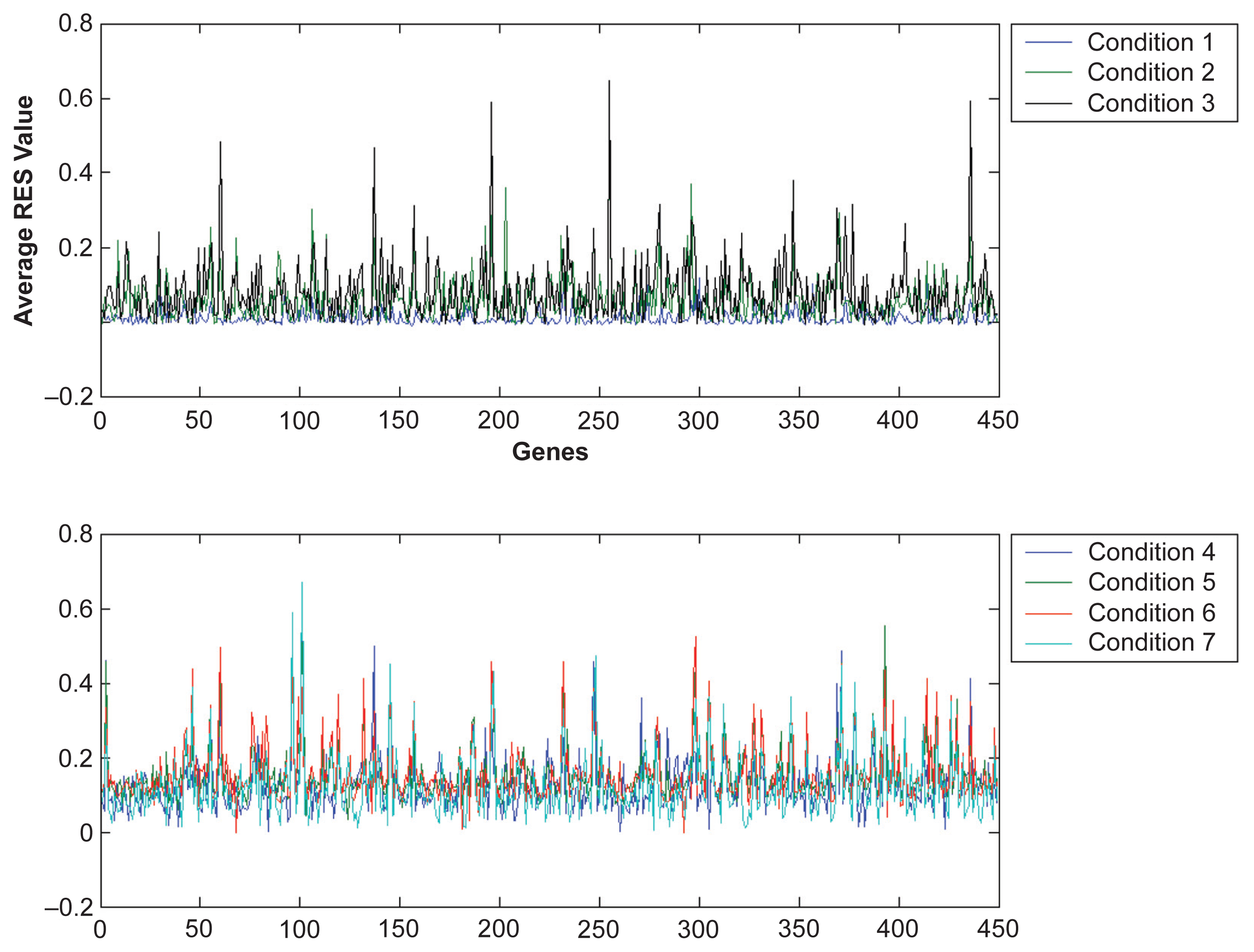

Supplement: Figure S4 — Averaged REV scores for different experimental conditions. [file grsb-2009-049f11.tif]
